# Folic Acid Preconditioning Alleviated Radiation-Induced Ovarian Dysfunction in Female Mice

**DOI:** 10.3389/fnut.2022.854655

**Published:** 2022-06-28

**Authors:** Qianyu Zhang, Zhifu Wei, Huinan Weng, Ye Chen, Jie Zhang, Shiwei Mei, Jiahui Wei, Xiulan Zhu, Yingqi Nong, Jianxing Ruan, Wenjuan Liu, Ruiqiong Zhou, Fang Wang, Yanni Xie, Junjiu Huang, Xiqian Zhang, Fenghua Liu

**Affiliations:** ^1^Department of Reproductive Health and Infertility, Guangdong Women and Children Hospital, Guangzhou, China; ^2^Jinan University, Guangzhou, China; ^3^Department of Gynaecology, The Affiliated Shunde Hospital of Jinan University, Foshan, China; ^4^Department of Radiation, Guangdong Women and Children Hospital, Guangzhou, China; ^5^MOE Key Laboratory of Gene Function and Regulation, State Key Laboratory of Biocontrol, School of Life Sciences, Sun Yat-sen University, Guangzhou, China

**Keywords:** folic acid, radiation, ovarian dysfunction, reproduction, female

## Abstract

Radiological therapy/examination is the primary source of artificial radiation exposure in humans. While its application has contributed to major advances in disease diagnosis and treatment, ionizing radiation exposure is associated with ovarian damage. The use of natural products, either alone or as an adjunct, has become increasingly common for reducing the side effects of radiological therapy during disease treatment. Herein, we explored the protective effect of folic acid (FA), a widely used B vitamin, against radiation-induced ovarian injury and its mechanism of action. Female mice with normal ovarian function were randomly divided into control, FA, radiation, and radiation + FA groups. The intervention strategy included daily intragastric administration of FA (5 mg/kg) for 3 weeks prior to radiation exposure. Mice in the radiation and radiation + FA groups received a single dose of 5 Gy X-ray irradiation. Changes in the estrous cycle were then recorded, and ovarian tissues were collected. Pathophysiological changes as well as reproductive and endocrine-related indexes were determined via H&E staining, immunohistochemistry, Western blot, and ELISA. The reproductive performance and emotional symptoms of animals were also monitored. Our results indicated that FA intervention effectively alleviated ovarian damage, leading to more regular estrous cycles, lesser impairment of follicular morphology and endocrine status, as well as greater germ cell preservation. Reduced levels of oxidative stress, inflammation, and enhanced DNA repair were associated these changes. FA pre-administration improved the reproductive performance, leading to higher pregnancy rates and greater litter sizes. Further, the anxiety levels of animals were significantly reduced. Our results indicate that FA pre-administration significantly alleviates radiation-induced ovarian damage in rodents, highlighting its potential as a protective strategy against radiation exposure in the female population.

## Introduction

Radiation is distributed throughout the environment, emanating from natural sources as well as anthropogenic activity. Cosmic rays constitute the main source of radiation exposure for humans in the natural environment, while medical radiation is the major source of artificial radiation (1, 2). Medical ionizing radiation (IR) is widely used for clinical diagnosis and cancer therapy. Thus, radiation exposure is generally unavoidable and is predicted to induce health hazards, with the ovaries being among the organs most susceptible to radiation-induced injury (3). Therefore, increasing attention has been directed toward minimizing ovarian radiotoxicity.

Radiotherapy represents a major strategy for the treatment of cervical cancer, endometrial cancer, rectal cancer, and other malignancies (4–6). Diagnostic applications of radiation, such as mammography, are the gold standard for diagnosing various diseases, such as breast cancer (7). While the application of radiation in diagnosis and treatment has improved disease management, radiation exposure has also been shown to have deleterious effects on various living organisms, including humans, with organs of the reproductive system being the most susceptible to radiation damage (8–11).

Ovarian germ cells are highly sensitive to radiation, with exposure, such as through radiotherapy, causing inflammation and oxidative stress, which in turn leads to germ cell apoptosis (12, 13). Ovarian follicles composed of germ cells and granulosa cells represent the basic functional units of the ovary (14). Radiation-induced reduction of the follicle reserve and follicle atresia adversely affect reproductive function and hormone secretion, compromising female fertility, with various accompanying symptoms that severely impact the physical and mental health of women. Radiation exposure-induced premature ovarian failure (POF)-like features have been widely reported and are receiving increasing attention (15, 16). Moreover, medical radiation poses potentially serious risks to the fertility of female patients as well as female medical practitioners. At present, strategies for mitigating radiation-induced ovarian damage mainly include hormonal regulation, cryopreservation of germ cells and ovarian tissues, assisted reproductive technology, stem cell transplantation, and pharmacological therapy. However, these are associated with various disadvantages, including side effects, high cost, poor compliance, and suboptimal efficacy (17, 18). Taken together, the prevention and restoration of ovarian function following radiation-induced damage represents a major challenge in the clinic.

Folic acid (FA) is a B vitamin that serves as a coenzyme in nucleic acid and amino acid metabolism, thus participating in a number of important biochemical reactions within cells. Mounting evidence has shown that FA has antioxidant and anti-inflammatory properties (19, 20). Further, it is widely utilized for improving female fertility in the clinic (21). The therapeutic effects of FA overlap with the adverse effects of radiation-induced ovarian damage (22). It has been established that compounds with antioxidative properties harbor radioprotective capacity (23, 24). However, whether FA could protect against radiation-induced ovarian damage remains unclear. The pharmacological properties of FA and the pathogenesis of radiation-induced injury suggest that the former has potential for alleviating radiation-induced ovarian injury. In the present study, we aimed to investigate whether prophylactic FA administration could alleviate ovarian irradiation-induced reproductive and endocrine-related dysfunction in female mice.

## Materials and Methods

### Animal Model and Treatment

Ten-week-old female C57BL/6 mice were obtained from Beijing Vitong Lihua Co., Ltd. The mice were kept in a controlled standard environment (23±1 °C, 12-h light/dark cycles), with free access to standard food and clean water. After basic physical examination and confirmation of regular estrous cycles (based on morphological changes in vaginal cytology smears obtained every morning at 8:30 am and observed under a microscope), female mice were randomly assigned to one of four groups: control group (Con), FA pre-administration control group (FA), radiation exposure group (RA), and FA pretreatment + radiation exposure group (RA+FA). FA pre-administration dosage and protocol were selected on the basis of our previous study (25). Briefly, FA (5 mg/kg; Sigma, St. Louis, MO, USA) was administered daily via gavage. Radiation exposure was performed after 3 weeks of FA pre-administration. Prior to radiation exposure, animals were anesthetized with 1% pentobarbital sodium (40 mg/kg). Only the ovarian parts of the mouse abdomen were fully exposed, while the rest was covered with lead plates. Local radiation exposure was carried out using X-rays from an Elekta Synergy linear accelerator (Elekta AB, Sweden) at a distance of 90 cm, and a single dose of 5 Gy (absorbed dose rate: 1.5 Gy/min) was administered (26). All animal experimental procedures were formally reviewed, approved, and supervised by the ethics committee of the Guangdong Women and Children Hospital (202101345). All efforts were made to minimize animal suffering.

### Ovarian Morphology Observation

At the end of experiments, mice were euthanized by cervical dislocation, and ovarian tissues were rapidly extracted. For hematoxylin and eosin (H&E) staining, tissues were fixed in 4% paraformaldehyde fixative solution for 24 h. After routine gradient dehydration and paraffin embedding, ovarian tissue samples were cut into 5-μm-thick sections and stained with H&E and terminal deoxynucleotidyl transferase dUTP-mediated nick-end labeling (TUNEL) staining (C1090, Biyuntian, China). A microscope (Olympus DP71, Tokyo, Japan) was used to capture images of the sections, and follicles were then quantified using CaseViewer software (3D Histech Ltd., Budapest, Hungary).

### ELISA

Freshly collected blood or ovarian tissue was homogenized and/or centrifuged at 11,000 × *g* and 4 °C for 10 min. The supernatant was collected to determine the levels of follicle-stimulating hormone (FSH, J&L Biological), luteinizing hormone (LH, J&L Biological), progesterone (P, Beyotime), estradiol (E, Beyotime), anti-Mullerian hormone (AMH, J&L Biological), superoxide dismutase (SOD, KeyGEN BioTECH), malondialdehyde (MDA, KeyGEN BioTECH), glutathione peroxidase (GSH-Px, KeyGEN BioTECH), and reactive oxygen species (ROS, KeyGEN BioTECH) using commercially available kits. The absorbance of each sample was read at 450 nm using a microplate reader, and each experiment was repeated independently in triplicate.

### Immunohistochemistry

The ovarian tissues were cut into 5-μm-thick sections. After antigen retrieval, the ovarian sections were incubated overnight with a rabbit polyclonal antibody against AMH (ab103233, 1:2000, Abcam). After washing, the immunoperoxidase-labeled Donkey Anti-Rabbit IgG secondary antibody (1:2000, LS-C60950, LSBio) was applied to all slides at room temperature for 1 h. After washing with PBS three times, DBA was used for color rendering, and the nuclei were re-stained with hematoxylin for 2 min. Finally, the slides were sealed with neutral adhesive for observation, and the AMH signals were acquired using a light microscope (BX51, Olympus, Tokyo, Japan).

### Immunofluorescence

Formalin-fixed, paraffin-embedded ovarian tissues were sectioned to a thickness of 4 μm. After routine deparaffinization and rehydration, the sections were blocked using 3% bovine serum albumin and incubated with a primary anti-MVH (ab13840, 1:500, Abcam) antibody overnight at 4 °C, followed by incubation with an Alexa Fluor-594-conjugated secondary antibody (Invitrogen). The samples were co-stained with 4',6-diamidino-2-phenylindole (DAPI) and examined using a confocal microscope (LSM510, Carl Zeiss, Goettingen, Germany).

### Immunoblotting Analysis

Ovarian tissues were homogenized in RIPA buffer (Thermo Scientific, Rockford, IL, USA) and centrifuged at 12,000 × *g* for 20 min. The protein content of samples was determined using a BCA protein assay kit (Bio-Rad Laboratories, Carlsbad, CA). Aliquots of each sample were separated via 12% SDS-PAGE and then transferred onto a nitrocellulose membrane, which was blocked with non-fat milk for 1 h. The membranes were probed overnight at 4°C with antibodies against the following proteins: HO-1 (ab68477; 1:2000; Abcam); NRF2 (ab92946; 1:3000; Abcam), TNF-α (SAB5700627; 1:1000; Sigma), IL-1β (AB1413-I; 1:4000; Sigma), IL-6 (SAB5700632; 1:2000; Sigma); MVH (ab13840; 1:3000; Abcam); OCT4 (ab184665; 1:2000; Abcam); Ki67 (ab16667; 1:4000; Abcam), PCNA (mAb2586; 1:3000; Cell Signaling Technology), ATM (ab199726; 1:2000; Abcam), RAD51 (ab176458; 1:3000; Abcam), and GAPDH (ab181602; 1:3000; Abcam). After washing with PBST buffer solution three times, the membranes were incubated with appropriate horseradish peroxidase-conjugated secondary antibodies (Beyotime Institute of Biotechnology) for 1 h at 25 °C, and the protein bands were visualized using an enhanced chemiluminescence kit (Thermo Fisher Scientific Inc.). The blots were scanned and normalized to GAPDH for quantification.

### Open Field Test

The space exploration ability and anxiety-like behavior of mice were evaluated in the open field test. The mice were acclimated to the laboratory environment for 30 min prior to the experiment. As previously described, the mice were gently placed at the center of an open box and allowed to explore the environment freely for 5 min. The spontaneous activity of mice within 5 min was recorded and evaluated using activity analysis software (Ethovision 7.0, Noldus, Wageningen, Netherlands). Acquired data included the total traveled distance and the stay time of mice in the central grid. After testing each mouse, the experimental device site was wiped with 75% alcohol to avoid odor interference in all behavioral experiments.

### Elevated Plus Maze Test

Anxiety-like behavior was examined using the elevated plus maze as previously described (27). The maze device consisted of two crossed open arms (30 × 6 cm) and two crossed closed arms (30 × 6 × 25 cm) with a square center open platform (6 × 6 cm). During the experiment, mice were placed on the central platform facing the open arm. The number of open/closed arm entries and time spent in open/closed arms were recorded separately and used as indicators to evaluate anxiety.

### Statistical Analysis

GraphPad PRISM version 6 (GraphPad Software, Inc, La Jolla, CA, USA.) was used for statistical analysis and illustration. All data were expressed as the mean ± standard error of mean (SEM). One-way analysis of variance (ANOVA) was used to assess the differences between multiple groups, followed by a Bonferroni's *post hoc* test. A *P* < 0.05 indicated statistical significance.

## Results

### Protective Effects of FA Pre-administration on Radiation-Induced Menstrual Cycle Disturbance and Ovarian Atrophy

**Figure 1:**
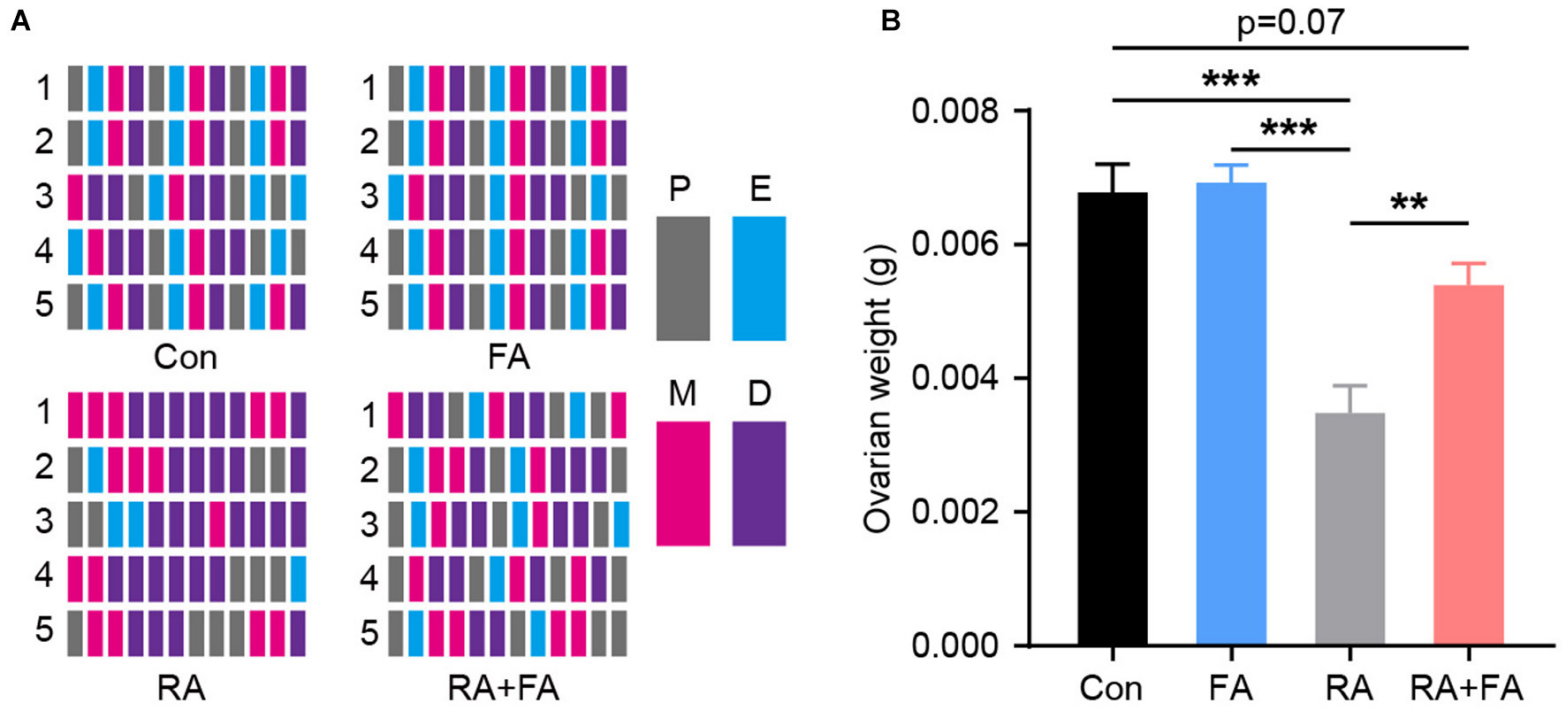
Effect of folic acid pre-administration on menstrual cycle and ovarian atrophy after radiation injury. **(A)** Raster plot schematic representing the menstrual cycle following injury due to radiation exposure. Representative raster plots were selected from five mice in each group which were closest to the mean levels. P, proestrus; E, estrus; M, metestrus; D, diestrus. **(B)** Quantification of the ovarian weight for each group. Error bars, standard error of mean (SEM). *N* = 8, ^**^p < 0.01 and ^***^p < 0.001.

The estrous cycle was evaluated based on vaginal smear slides stained with crystal violet immediately after RA injury. In terms of regularity, mice in the Con and FA groups had a similar regular menstrual cycle. However, irregular menstrual cycles were observed in the RA group; this could be reversed by FA pre-administration which improved cycle regularity (Figure 1A). The ovarian weight was similar between the Con and FA groups. However, the ovarian weight of mice in the RA group was significantly decreased (Con: 0.006775 ± 0.00043 g, FA: 0.006925 ± 0.0002644 g, RA: 0.003475 ± 0.0004113 g, RA+FA: 0.005388 ± 0.0003303 g). Thus, FA pretreatment significantly prevented ovarian atrophy compared to radiation alone (Figure 1B).

### FA Pre-administration Alleviates Radiation-Induced Ovarian Follicle Damage

**Figure 2:**
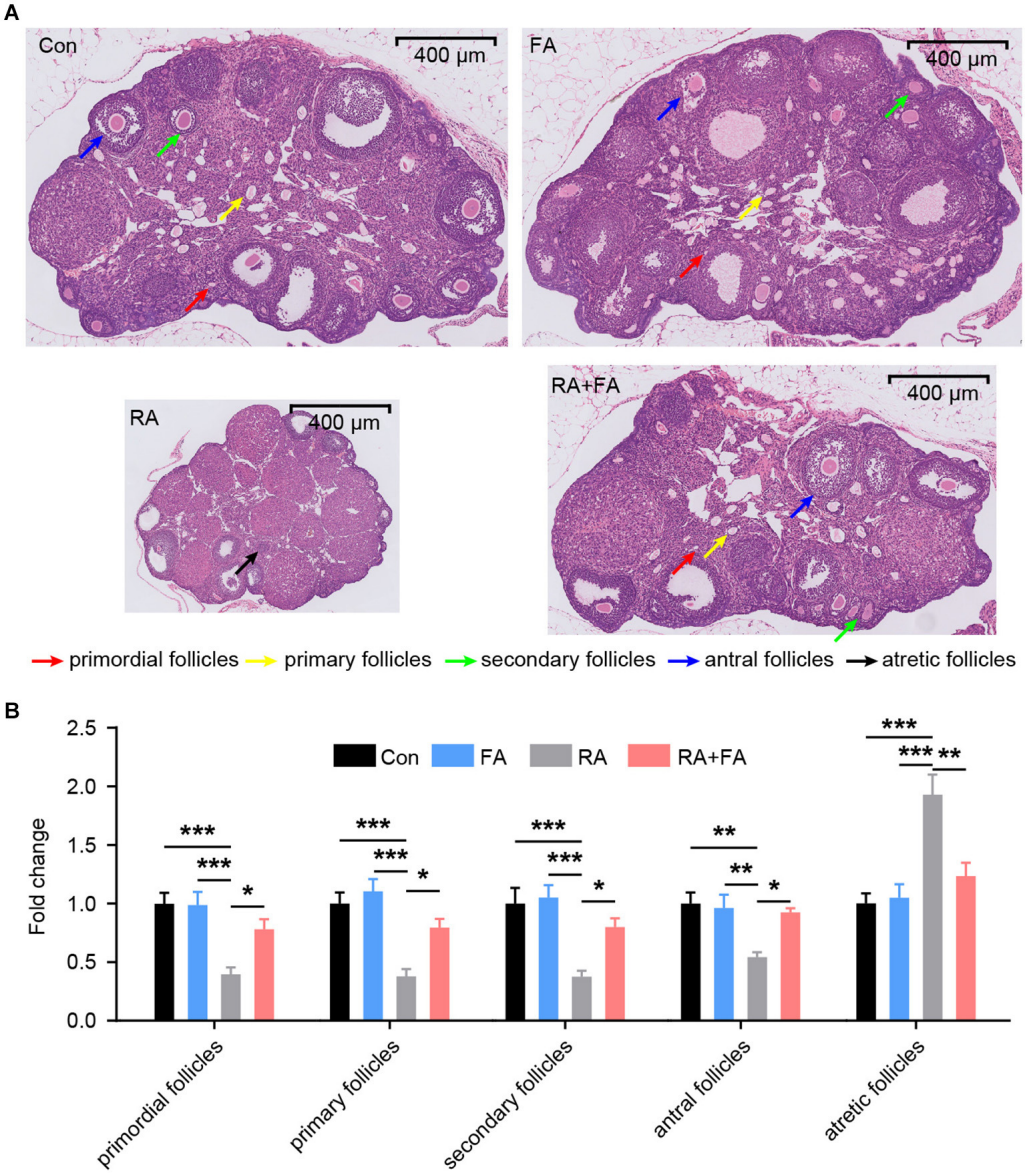
Effect of folic acid (FA) pre-administration on ovarian pathological damage after radiation injury. **(A)** The results of hematoxylin and eosin (H&E) staining indicated that FA pre-administration relieved ovarian histopathological damage. Red arrow: primordial follicles; yellow arrow: primary follicles; green arrow: secondary follicles; blue arrow: antral follicles; black arrow: atretic follicles. Scale bar, 400 μm. **(B)** Relative quantification of the ovarian follicles for each group. There was no significant difference in follicle number between the Con group and FA group (*p* > 0.05). The numbers of primordial follicles, primary follicles, secondary follicles, and antral follicles in the RA group were significantly lower than those in the Con group. FA pre-administration significantly relieved the effect of radiation on the ovarian tissue structure and increased the number of ovarian follicles, thus enhancing ovarian reserve capacity. Con: control group, FA, FA pre-administration control group; RA, radiation exposure group; RA+FA, FA pretreatment + radiation exposure group. Error bars, standard error of mean (SEM). *N* = 7, ^*^p < 0.05, ^**^p < 0.01, and ^***^p < 0.001.

We then assessed morphological changes in ovarian tissue via H&E staining which revealed that ovarian tissues of the Con and FA groups exhibited normal morphology without pathological changes (Figure 2A). The structure of ovarian tissues in the Con and FA groups was normal, and the follicles at various stages of development as well as corpus lutei were abundant. After radiation, the ovaries exhibited atrophy. In the RA group, the number of follicles at nearly all stages were decreased, and the number of atretic follicles was increased, accompanied by fibrosis in the stroma. Compared with the RA group, the FA + RA group exhibited obvious improvement, that is, the total number of female follicles increased, a large number of primordial, primary, secondary, and antral follicles were preserved, with fewer atretic follicles present (Figure 2B). Thus, FA pre-conditioning alleviated ovarian follicle damage after radiation exposure.

### Effect of FA Pre-administration on Radiation-Induced Reproductive Hormone Dysregulation

**Figure 3:**
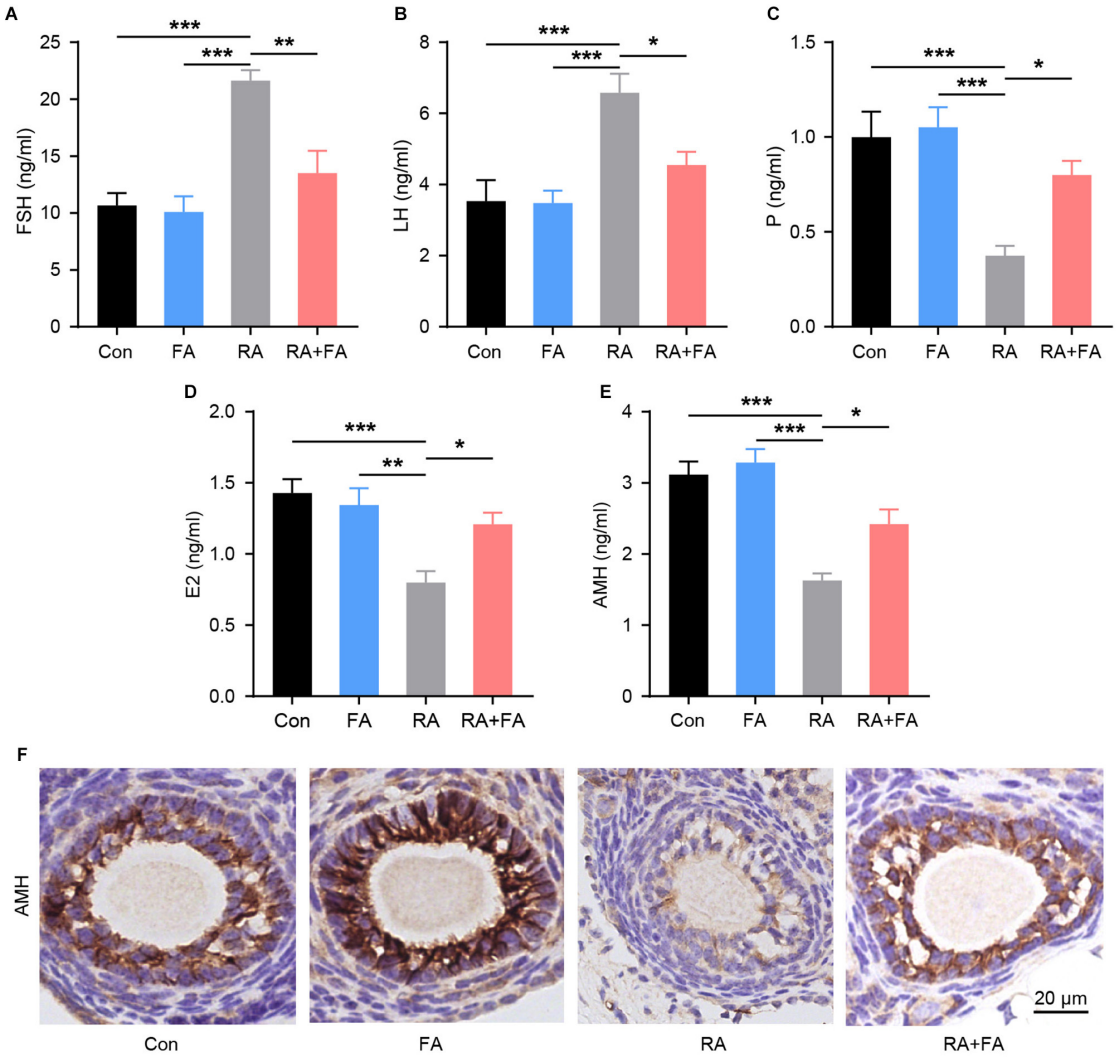
Radiation-induced dysregulation of reproductive hormone secretion was alleviated via folic acid (FA) pre-administration. **(A–E)** Effect of FA pre-administration on serum FSH, LH, P, E2, and AMH levels determined via ELISA. FSH, follicle-stimulating hormone; LH, luteinizing hormone; P, progesterone; E2, estradiol; AMH, anti-Mullerian hormone. Each ELISA assay was repeated three times. **(F)** Immunohistochemical staining for AMH in ovarian follicles of each group. Con, control group; FA, FA pre-administration control group; RA, radiation exposure group; RA+FA, FA pretreatment + radiation exposure group. Scale bar, 20 μm. Error bars, standard error of mean (SEM). *N* = 7–8, ^*^p < 0.05, ^**^p < 0.01, and ^***^p < 0.001.

Since the ovaries are responsible for the synthesis and secretion of hormones that regulate reproductive function, we examined the levels of serum sex hormone markers and ovarian reserve markers after radiation. Compared with the Con group, the serum levels of FSH and LH were markedly increased upon radiation, with FA pre-administration lowering both (Figures 3A,B). Moreover, the concentrations of P, E2, and AMH in the RA group were lower than those in the Con group. These hormones were upregulated in the RA + FA group relative to the RA group (Figures 3C–E). Similarly, AMH immunohistochemistry staining in ovarian tissue indicated that irradiation decreased the AMH-positive area relative to the Con group (Figure 3F). Conversely, FA pre-administration upregulated AMH signal density relative to the RA group.

### Protective Effect of FA Pre-administration Against Radiation-Induced Oxidative Stress and Inflammatory Injury of Ovarian Tissue

**Figure 4:**
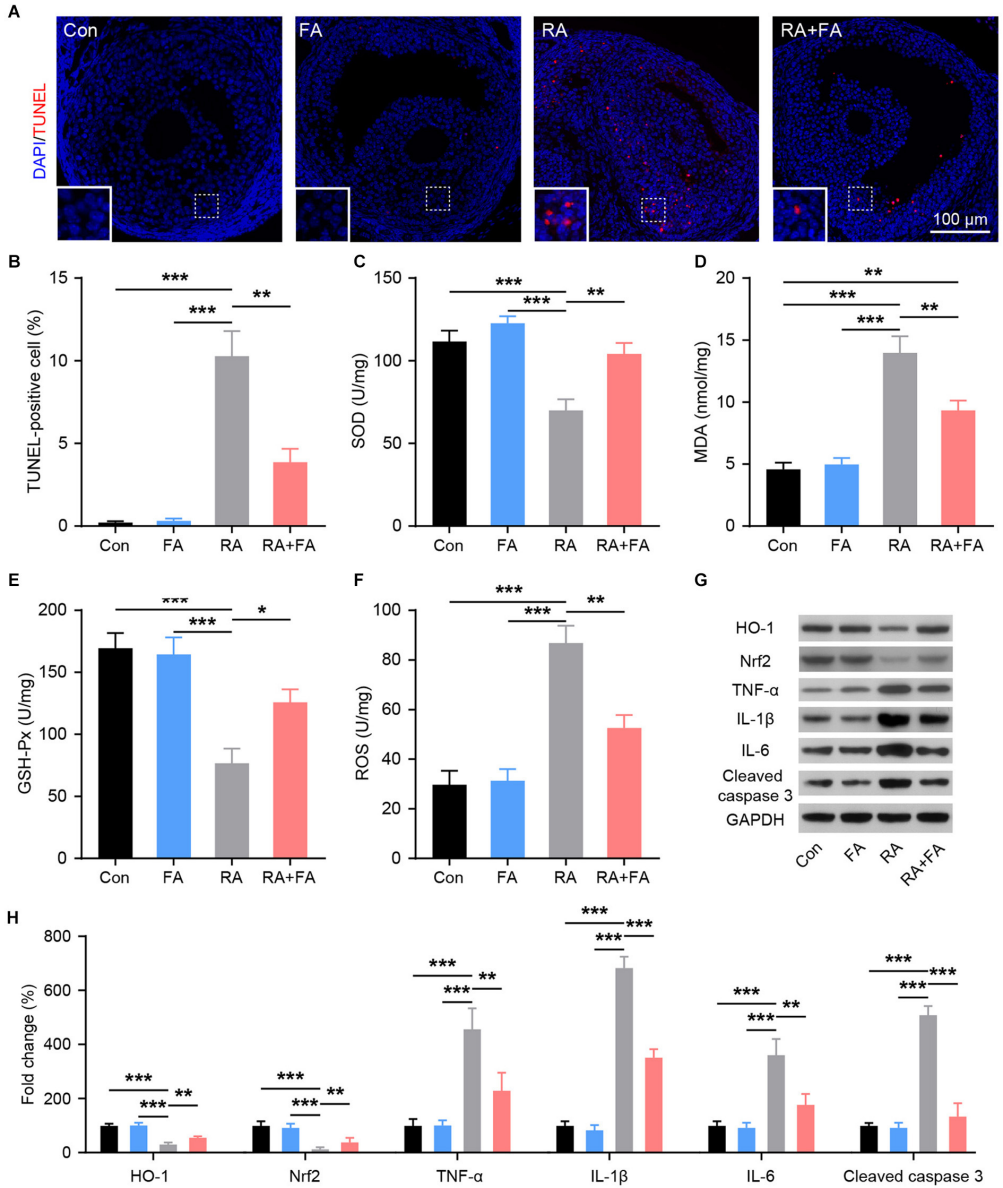
Effect of folic acid (FA) pre-administration on radiation-induced ovarian apoptosis, oxidative stress, and inflammation. **(A,B)** Ovarian follicle cell apoptosis was determined via TUNEL staining. Apoptotic cells are stained in red, and nuclei are stained in blue (DAPI). Scale bar, 100 μm. **(C–F)** Effect of FA pre-administration on the levels of SOD, MDA, GSH-Px, and ROS in ovarian homogenates. SOD, superoxide dismutase; MDA, malondialdehyde; GSH-Px, glutathione peroxidase; ROS, reactive oxygen species. Each ELISA assay was repeated three times. **(G,H)** Western blot was performed to detect the protein levels of HO-1, Nrf2, TNF-α, IL-1β, IL-6, and Cleaved caspase-3 in ovarian tissues from each group.in ovarian tissues from each group. HO-1, heme oxygenase 1; Nrf2, nuclear factor-erythroid 2-related factor 2. TNF-α, tumor necrosis factor-α; IL-1β, interleukin-1β; IL-6, interleukin-6; Con, control group; FA, FA pre-administration control group; RA, radiation exposure group; RA+FA, FA pretreatment + radiation exposure group. Error bars, standard error of mean (SEM). *N* = 8, ^*^p < 0.05, ^**^p < 0.01, and ^***^p < 0.001.

To evaluate the protective mechanism of FA on ovarian viability, TUNEL staining was used to assess ovarian apoptosis. Increased ovarian follicle apoptosis was observed after radiation injury, while FA pre-administration effectively decreased the number of TUNEL-positive cells in the ovarian tissue (Figures 4A,B). The levels of factors related to oxidative stress (SOD, MDA, GSH-Px, and ROS) in ovarian tissue homogenates were analyzed using ELISA or western blot. As shown in Figures 4C–F, radiation downregulated the activity of SOD and GSH-Px while upregulating MDA and ROS levels. Compared with the RA group, FA pre-administration significantly upregulated SOD and GSH-Px, while downregulating MDA and ROS levels. Nrf2/HO-1 is an intracellular system driving the antioxidant response. HO-1 and NRF2 levels were significantly lower in the RA group than in Con group. Further, the expression of NRF2 and HO-1 was effectively maintained in the RA+FA group following irradiation (Figures 4G,H). Western blot results further confirmed that radiation injury dramatically upregulated the oxidative stress response as well as inflammatory cytokines, TNF-α, IL-1β, and IL-6, while FA pre-administration alleviated these effects (Figures 4G,H).

### Molecular Mechanism Underlying the Protective Effect of FA Pre-administration Against Radiation-Induced Ovarian Injury

**Figure 5:**
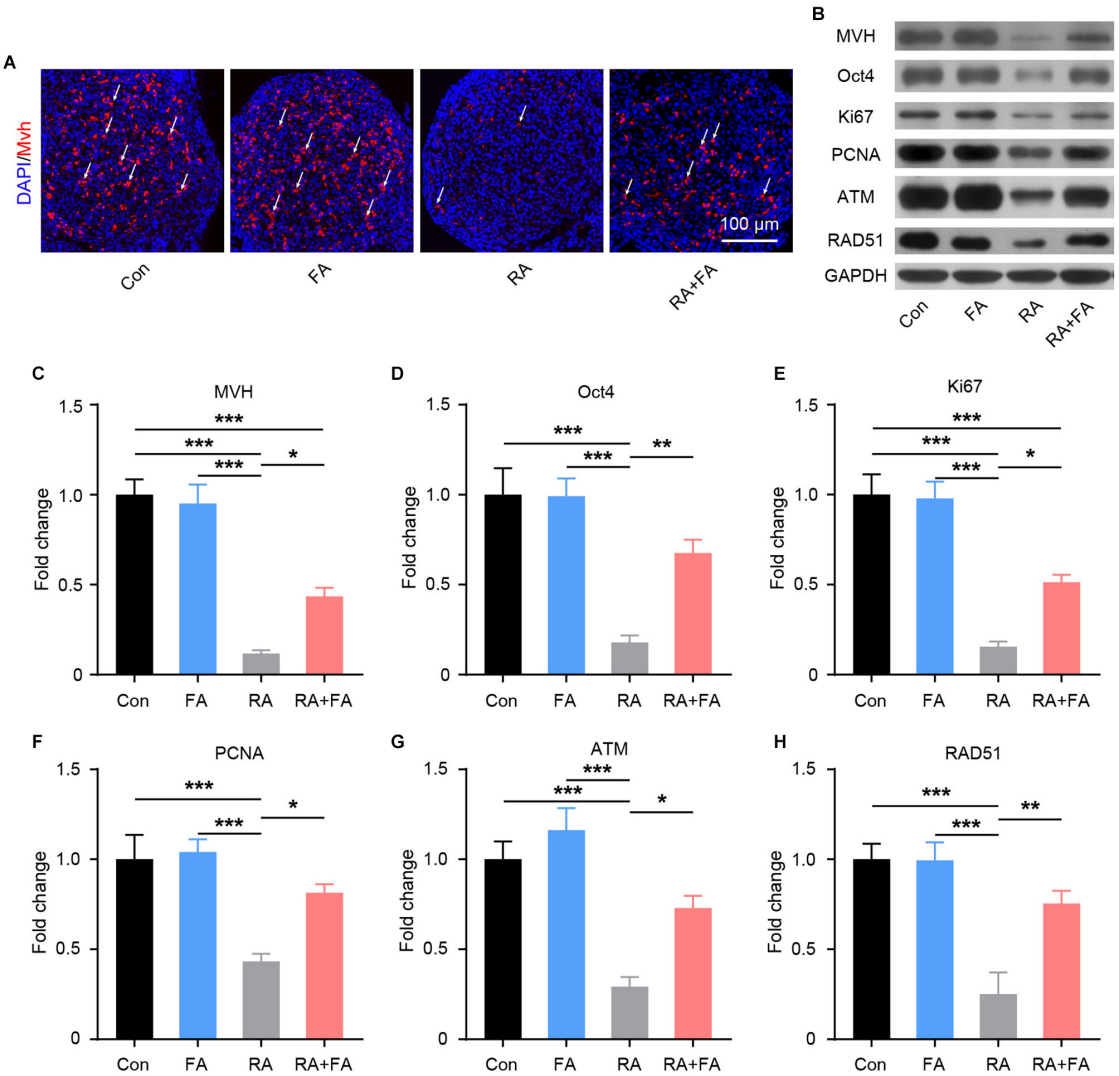
Folic acid (FA) pre-administration reverses the radiation-induced suppression of reproductive ability in female mice. **(A)** MVH-positive ovarian germ cells were observed via immunofluorescence analysis of ovarian tissue. Germ cells are stained in red, and nuclei are stained in blue (DAPI). Scale bar, 100 μm. **(B)** Western blot was performed to detect the protein levels of MVH, Oct4, Ki67, PCNA, ATM, and RAD51 in ovarian tissues from each group. MVH, mouse vasa homolog; PCNA, proliferating cell nuclear antigen; ATM, ataxia-telangiectasia mutated. **(C–H)** Immunoblot semi-quantitative analysis of MVH, Oct4, Ki67, PCNA, ATM, and RAD51 in ovarian tissues of each group. Error bars, standard error of mean (SEM). *N* = 8, ^*^p < 0.05, ^**^p < 0.01, and ^***^p < 0.001.

Given the importance of germ cells for normal ovarian function, we examined the effects of radiation exposure on germ cells within ovarian tissues. As shown in Figure 5A, the germ cell marker mouse vasa homolog (MVH) was visualized via immunofluorescence staining. Radiation exposure induced a marked decrease in MVH-positive cells, while FA pre-administration preserved germ cells relative to the RA group. These results were confirmed via quantitative immunoblot analysis (Figures 5B,C). As ovarian germline stem cells are radiosensitive, we assessed the effect of FA on the expression of Oct4, a major transcription factor for ovarian stem cell self-renewal. Oct4 expression was reduced in mice with radiation-induced ovarian injury, which was reversed via FA pre-administration (Figure 5D). Immunoblot analysis of Ki67 and PCNA further demonstrated that FA pre-administration attenuated radiation-induced suppression of ovarian cell proliferation (Figures 5B,E,F). Accordingly, we hypothesized that the recovery of ovarian cell proliferation under FA pre-administration was due to enhanced DNA repair. To assess this, we examined the expression of DNA repair-associated proteins. Western blot analysis indicated that FA pre-administration markedly upregulated the protein expression of DNA repair markers ATM and RAD51 upon irradiation (Figures 5B,G,H). Taken together, these results indicated that FA pre-administration reverses the radiation-induced negative effect on female mouse reproductive ability by enhancing DNA repair capacity and thus improving germ cell radioresistance.

### FA Pre-administration Alleviates Radiation-Induced Reproductive Dysfunction in Female Mice

**Figure 6:**
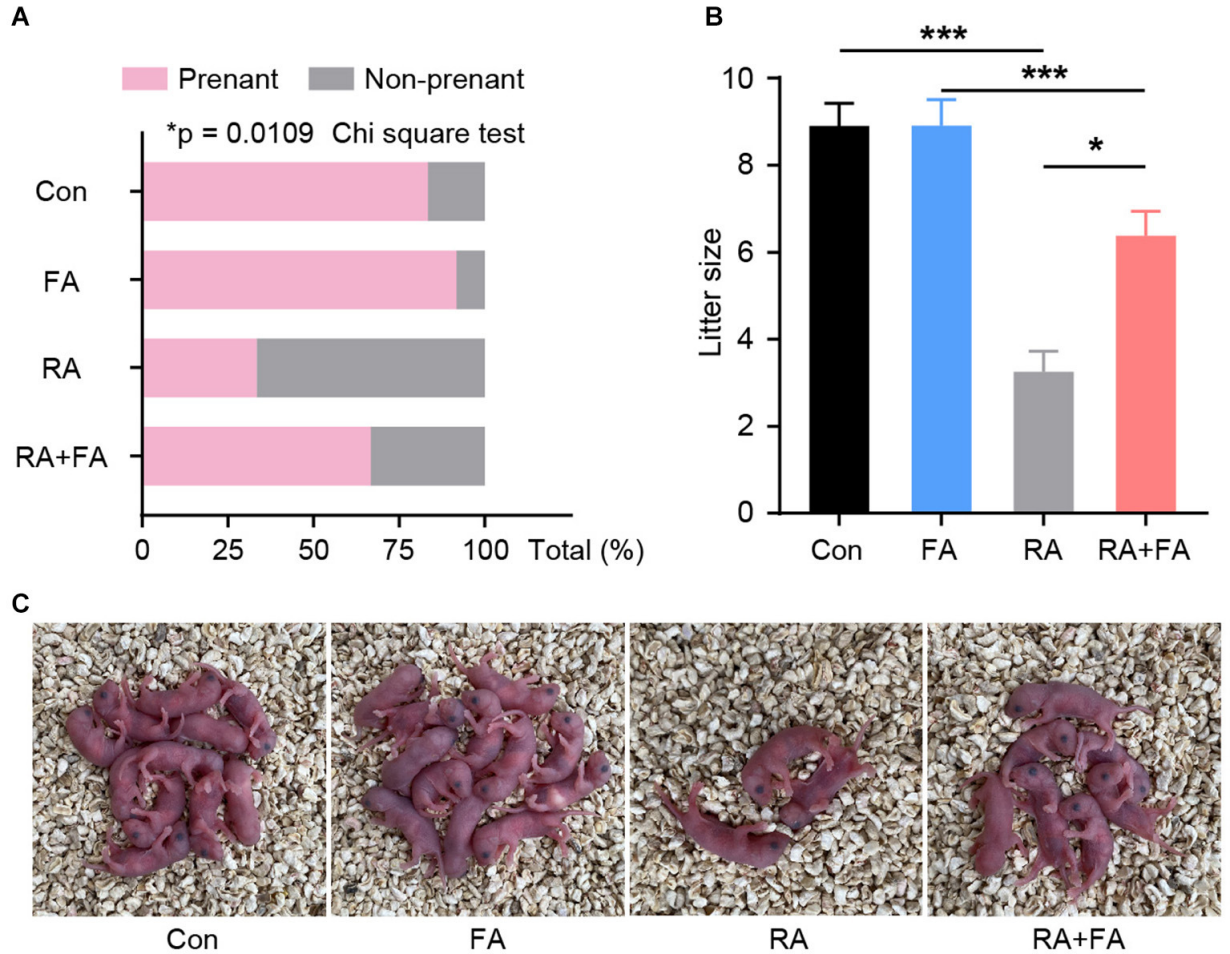
Effect of folic acid (FA) pre-administration on reproductive performance after radiation injury. **(A)** Quantitative analysis of the pregnancy rate in each group. *N* = 12, ^*^p < 0.05, Chi square tes. **(B)** Quantitative analysis of the average litter sizes produced by the female mice of each group. ^*^p < 0.05 and ^***^p < 0.001. **(C)** Representative pictures of the litter at birth for each group. Con, control group; FA, FA pre-administration control group; RA, radiation exposure group; RA+FA, FA pretreatment + radiation exposure group. Error bars, standard error of mean (SEM).

Our results prompted us to further determine whether FA pre-administration has a beneficial effect on animal reproductive performance. Male mice with normal fertility were mated with each group of females. The pregnancy rate among mice in the RA group was significantly lower than that in the Con and FA groups (both over 80%) (Figure 6A), with more than 66% of female mice in the RA group experiencing unsuccessful pregnancies. The pregnancy rate among female mice pre-administered FA was about 66%. In addition, the litter size of mice in the RA group was significantly reduced, but this effect was effectively reversed via FA pre-administration (Figures 6B,C).

### FA Pre-administration Relieves Ovarian Irradiation-Induced Emotional Disturbance

**Figure 7:**
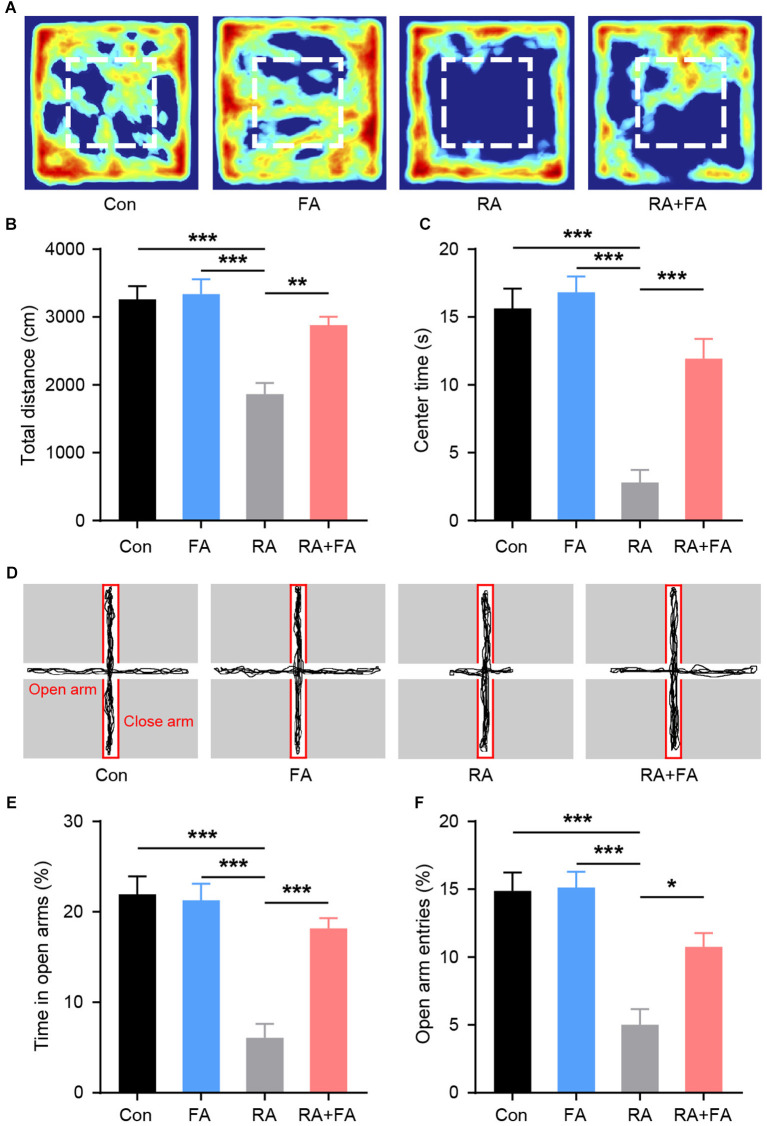
Effect of folic acid (FA) pre-administration on emotional behaviors observed in female mice after ovarian radiation injury. **(A)** Heat map of the exploration trajectories representing the activity of mice in each group. Representative heat maps were selected from the mouse which was closest to the mean level for each group. Red boxes indicate center area. **(B)** Quantification of the total distance traveled for each group. **(C)** Quantification of center area exploration time for each group. **(D)** Representative track of elevated plus maze exploration for each group. **(E)** Time spent in the open arm of the elevated plus maze. **(F)** Entries into open arms of elevated plus maze. Error bars, standard error of mean (SEM). *N* = 8, ^*^p < 0.05, ^**^p < 0.01, and ^***^p < 0.001.

Patients undergoing ovarian radiation are known to experience emotional symptoms (28). Therefore, we examined the emotional behaviors in each group. During the open field test (Figure 7A), there were no significant differences in the distance traveled between groups (Figure 7B). However, radiation-exposed mice exhibited a significantly shorter time in the center area of the arena. FA pre-administration increased center area exploration relative to that of RA group mice (Figure 7C). The lower center activity indicated an increased level of anxiety among RA group animals. Assessment of anxiety-like behavior with an elevated plus maze was then carried out. We observed that radiation-exposed mice spent less time exploring the open arm than Con mice (Figures 7D–F), suggesting increased anxiety-like behavior in the former. In contrast, FA pre-administration significantly increased open-arm entries and exploration time, suggesting an anxiolytic effect in mice that underwent ovarian irradiation.

## Discussion

Considering the increasing incidence of gynecological and gastrointestinal tumors, a greater number of female patients undergo radiotherapy, which is a well-established treatment approach for various malignancies. While these female patients experience significantly improved treatment outcomes and life expectancy, they are under an increased risk of radiation damage to adjacent organs, with the ovaries being among the most common sites of radiation-induced injury. Women who suffer from ovarian radiation damage may develop secondary conditions such as amenorrhea, infertility, and premature ovarian failure, which severely affects their physical and mental health. Thus, minimizing the side effects of radiation therapy remains an unsolved clinical challenge. China has the largest female population in the world, which implies that a great number of Chinese women suffering potential risks for radiation-related ovarian damage, highlighting the urgent need for effective protection methods against radiation.

Radiation, such as in the form of X-ray irradiation, exhibits strong tissue penetration (29). Although physical protection and radiation dosage control are effective strategies to reduce radiation damage, they have certain limitations regarding operability, maneuverability, objectivity, and repeatability. Therefore, the focus should shift toward strategies based on drug intervention, while considering the adverse effects of potential therapeutics. FA is a physiologically essential vitamin and has been found to reduce side effects due to treatment in multiple organs (30, 31). Herein, we report for the first time the protective effect of FA pre-administration on radiation-induced ovarian injury. We demonstrated that FA pre-administration protected female mice against radiation-induced endocrine and reproductive dysfunction.

Clinical studies have identified radiation exposure as a major risk factor for premature ovarian failure (32). It has a strong detrimental effect on DNA synthesis (33). Further, radiation induces oxidative stress, inflammation, and the subsequent apoptosis of germ cells. Normal ovarian function depends on the continuous growth and development of follicles. Radiotherapy not only kills proliferating tumor cells, but also leads to the apoptosis of germ cells, thus causing follicle failure. Previous studies have demonstrated that FA has antioxidant and anti-inflammatory effects, suggesting that it may hold potential for the mitigation of radiation damage in the ovaries (23, 24). However, the effect of FA on radiation damage has remained unclear. Therefore, in this study, we established a mouse ovarian radiation injury model and explored the protective effect of FA pre-administration against radiation-induced ovarian injury. We first examined apparent disturbance of the estrous cycle following ovarian injury. Animals exhibited irregular estrous cycles following a single moderate dose of X-ray radiation, which further confirmed damage to the female reproductive system caused by ionizing radiation. This is consistent with previously reported ovarian dysfunction symptoms of menstrual irregularity (34). Animals in the FA pre-administration group had more regular menstrual cycles, suggestive of a protective effect of FA on ovarian function. This supports our hypothesis that FA pre-administration may be a potential strategy for mitigating ovarian radiation damage.

Pathological and morphological observation results revealed atrophy of ovarian tissue and a significant reduction in the number of follicles following radiation exposure, which was consistent with the findings of previous reports (12, 13). In this study, we showed that FA-pretreated animals exhibited significantly lower ovarian tissue atrophy, more follicles were reserved, and there was a reduced occurrence of atresia. The reserved follicle is the basic functional unit of the ovary for reproduction and endocrine regulation. Further, the existence of oocytes and granulosa cells in follicles is the physiological basis of reproductive and endocrine follicular function (35, 36). Follicular atresia, which is observed during ovarian injury, is the result of ovarian granulosa cell and oocyte apoptosis, eventually leading to symptoms of premature ovarian failure. Our experimental results indicated that, compared with mice of the radiation group, those receiving FA pre-administration had significantly improved serum reproductive hormone levels and better ovarian endocrine function, suggesting that FA could alleviate the follicular damage caused by radiation. Correspondingly, FA pre-administration remarkly reduced the number of TUNEL-positive apoptotic cells within follicles. Oxidative stress is one of the main mechanisms of radiation injury and leads to cell apoptosis (37). Nrf2 is the most active among leucine zipper transcription factor, driving the downstream expression of genes that encode antioxidant proteins to facilitate the body's defense against oxidative stress and to protect cells against radiation (38). The expression of HO-1 has been widely demonstrated to have anti-inflammatory and antioxidant effects (39). FA pre-administration upregulated the expression of various antioxidant response-related molecules, including HO-1, in the ovaries and significantly suppressed oxidative stress and inflammation levels caused by radiation exposure, thus playing a role in alleviating radiation-induced damage. This is consistent with the previously reported effect of FA on spleen injury induced by lead acetate and cardiac dysfunction induced by hyperhomocysteinemia (40, 41).

The suppression of oxidative stress and inflammation contributes to the preservation of functional cells within the ovaries. Immunohistochemistry analysis confirmed that FA pre-administration resulted in a significant increase in preserved reproductive cells within the ovaries. In view of the effects of FA on DNA synthesis and repair, we explored whether pre-administration could promote DNA repair (42). ATM and its downstream factor RAD51 are key molecules involved in DNA repair (43). We found that FA pre-administration upregulated the expression of both ATM and RAD51. This provided a basis for the specific mechanism underlying the protective effect of FA in damaged follicles. Taken together, the current findings support the potential of FA in mitigating reproductive impairment in animals.

We examined the actual reproductive performance of animals using two main indicators, namely pregnancy rate and litter size, which reflect ovarian and follicular function at two levels. Most female animals in the control and FA groups became pregnant within four weeks of co-caging with males of normal reproductive function. Pregnancy rates were significantly reduced in the animals exposed to radiation. We suspect that the ovarian impairment-induced POF-like hormonal state suppresses the reproductive function of animals, reducing their libido and leading to lower reproductive performance. Litter size, on the other hand, is an indicator of the number of functional follicles within the ovary. Damage to the ovarian follicles reduces their ability to bind to sperm. The observed litter sizes were consistent with the follicular morphology, confirming the protective effect of FA pre-administration against radiation-induced impairment of ovarian reproductive function.

Radiation exposure-associated emotional disturbances are common and manifest as anxiety and depression, among other symptoms (44, 45). Therefore, we examined emotion-related symptoms in the present study. The results indicated that female mice subjected to ovary irradiation exhibited significantly increased levels of anxiety. These observations were similar to the symptoms observed in patients with POF (46). Previous studies have shown that radiation can cause mood changes in animals (44). In the present study, we covered other parts of the body to exclude damage to organs other than the ovaries. Thus, we report for the first time the direct relationship between radiation damage to the ovaries and mood change. A previous report demonstrated that FA can regulate emotions in women (25). We further characterized the regulatory role of FA in radiation-induced mood abnormalities. Our findings suggested that FA significantly alleviated the increased anxiety caused by ovarian radiation exposure, which may be related to the improvement of peripheral hormone levels promoted via FA pre-administration.

Our study fully considered and implemented the 3R principle in animal studies, and, although we have preliminary evidence for the protective effect of FA against radiation-induced ovarian damage, several important questions remain to be answered. First, the optimal combination and dose for intervention strategies remain to be determined. As a measure of reproductive function, the pregnancy rate indicates the percentage of females with bulging bellies who give birth to pups. Related factors, such as abortion, were not considered and studied in this process, which must be addressed in future studies. In addition, we did not examine the later development of progeny. Although we observed the activation of DNA repair mechanisms in the ovary, the protective effect of FA on radiation teratogenesis remains to be explored.

## Conclusions

In summary, the results of the present study further enhance our understanding of radiation-induced ovarian damage pathogenesis and progression. The present study demonstrated that FA pre-administration can effectively alleviate the ovarian damage induced by radiation in female mice, restore ovarian function, and relieve symptoms such as premature ovarian failure. Taken together, we offer a novel potential strategy for preserving ovarian function in female populations with potential radiation exposure risks.

## Data Availability Statement

The raw data supporting the conclusions of this article will be made available by the authors, without undue reservation.

## Ethics Statement

The animal study was reviewed and approved by Ethics Committee of Guangdong Women and Children Hospital.

## Author Contributions

All authors listed have made a substantial, direct, and intellectual contribution to the work and approved it for publication.

## Funding

This study was supported by grants from the Ministry of Science and Technology of the Republic of China (Project number: 2018YFC1002604), Guangdong Province Weiji Medical Develop's Fund (Project number: K-202104-2), Guangzhou Science and Technology Project (Project number: 202102080367), and Medical Science and Technology Research Fund of Guangdong Province (Project number: A202228).

## Conflict of Interest

The authors declare that the research was conducted in the absence of any commercial or financial relationships that could be construed as a potential conflict of interest.

## Publisher's Note

All claims expressed in this article are solely those of the authors and do not necessarily represent those of their affiliated organizations, or those of the publisher, the editors and the reviewers. Any product that may be evaluated in this article, or claim that may be made by its manufacturer, is not guaranteed or endorsed by the publisher.
